# Microbial and seminal traces of sexual intercourse and forensic implications

**DOI:** 10.1186/s40168-025-02268-7

**Published:** 2025-11-20

**Authors:** Sarah Ahannach, Thies Gehrmann, Irina Spacova, Stijn Wittouck, Jana Hiers, Peter A. Bron, Leonore Vander Donck, Maryse Cromphout, Meghna Swayambhu, Natasha Arora, Larissa Schuh, Iris Tournoy, Inge Smeers, Joke Wuestenbergs, Bram Bekaert, Ronny Decorte, Els Jehaes, Sarah Lebeer

**Affiliations:** 1 Laboratory of Applied Microbiology and Biotechnology, Department of Bioscience Engineering, University of Antwerp, Antwerp, Belgium; 2 U-MaMi Centre of Excellence, University of Antwerp, Antwerp, Belgium; 3 Department of Forensic Genetics, Institute of Forensic Medicine, University of Zurich, Zürich, Switzerland; 4 Laboratory of Forensic Genetics, Department of Forensic Medicine, University Hospitals Leuven, Leuven, Belgium; 5 Department of Forensic Medicine, University Hospitals Leuven, Leuven, Belgium; 6 Forensic Biomedical Sciences, Department of Imaging and Pathology, KU Leuven, Leuven, Belgium; 7 Forensic DNA Laboratory, Department of Forensic Medicine, Antwerp University Hospital, Edegem, Belgium

**Keywords:** Microbial forensics, Vaginal microbiome, Underwear microbiome, Sexual intercourse, Sexual assault, Semen, Biological trace evidence, Predictive modeling

## Abstract

**Background:**

The increasing numbers of sexual violence and unresolved rape cases require alternative approaches with higher evidential value to complement existing forensic tools. Predicting recent intercourse is crucial in forensic casework on sexual assaults. In this work, we assessed whether sexual intercourse can be predicted based on the vaginal microbiome and compared it to the gold standard method of semen detection.

**Results:**

Using a prediction model based on microbiome of 3043 women, intercourse was predicted with 71% accuracy in a balanced cross-validation machine learning setting. This prediction model was validated in a longitudinal intervention study and tested on forensic sexual assault cases. The developed predictor could accurately establish intercourse in 82% of the studied cases. Yet, underwear was found to hold an even greater evidential value and replace the more invasive vaginal sampling for semen detection in some cases with an accuracy of 95%. This was substantiated through a retrospective analysis of 207 forensic sexual assault cases.

**Conclusions:**

Taken together, this study revealed that the vaginal microbiome is better at predicting recent sexual intercourse, while the victim’s underwear has a clear value as additional biological trace evidence for semen detection. These findings are particularly useful in cases with delayed reporting and are obtained with less invasive sampling.

Video Abstract

**Supplementary Information:**

The online version contains supplementary material available at 10.1186/s40168-025-02268-7.

## Introduction

Approximately one-third of women worldwide have reported experiencing an act of sexual violence [1–3]. A lack of appropriate biological evidence (e.g., due to post-assault actions), no [4, 5] or delayed reporting (e.g., due to stigma and shock) and high dismissal rates (e.g., due to lack of evidence) [6] result in more than 90% of cases remaining unresolved [4, 7, 8]. Due to increasing appeals to “the right to remain silent” and contradictory testimonies [9], legal entities are no longer interested in only matching a trace to its donor (sub-source level; source/donor of a trace) but also in which actions would have led to the deposition of said trace (activity level) [10, 11]. Hence, in sexual assault cases, vaginal samples are very important for confirming penile penetration [12, 13]. Furthermore, semen detection in traces is a crucial step prior to DNA profiling to identify the donor. However, current techniques are limited to acid phosphatase (AP), microscopy, prostate-specific antigen (PSA or p30), and suspect DNA analysis [14], which disclose information at only the (sub-)source level [15, 16]. Alternative techniques such as microbiome forensics are available [17, 18], but studies and context-tailored data on vaginal microbiomes derived from sexual assault victims are currently lacking. The adult vaginal microbiome is typically dominated by one or two lactobacilli species or diverse anaerobes [19–21]. The penile and semen microbiomes have distinct compositions and harbor taxa such as *Pseudomonas*,* Prevotella*, and *Lactobacillus* [13, 22–24]. These microbes interact and may exchange during sexual intercourse, but microbial transfer has thus far mostly been studied, with a focus on sexually transmitted infections [24–29] and the exchange of pathogens [30, 31]. One study outside these clinical settings demonstrated that sexual intercourse increases bacterial diversity in semen and decreases *L. crispatus* diversity in vaginal samples, resulting in overall concordance between the microbes of partners [32]. However, how this microbial interchange impacts vaginal community dynamics is poorly understood, despite it being relevant for human reproduction and forensic casework. Moreover, vaginal swabs are often perceived as invasive, and their sampling should not be delayed [33]. Therefore, there is an urgent need for alternative or complementary biological evidence to add to the current forensic toolkit in sexual assault investigations, for instance, by incorporating textiles and, more specifically, underwear as sources of evidence in addition to intimate samples.

**Fig. 1:**
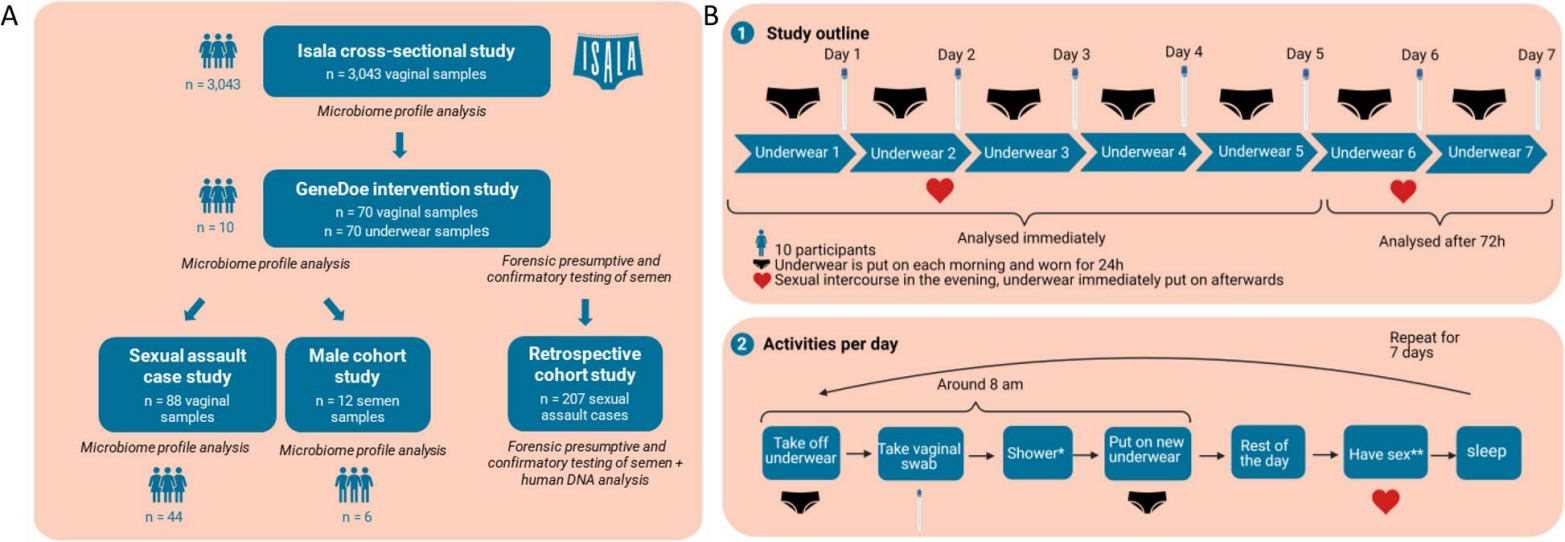
Schematic overview of **A **datasets and cohorts included in this study, with Isala (*n* = 3,043) as the mother dataset and **B **the GeneDoe study design and analyses. All participants (*n* = 10) wore seven pairs of underwear for consecutive days. Before each switch to the next pair, a vaginal swab was taken on the mornings. Participants had sexual intercourse exclusively on days 1 and 5. Underwear and vaginal swabs from days 1–5 were analyzed immediately, whereas those from days 6 and 7 were stored for 72 h at room temperature. *if preferred **on specific days

In this study, we investigated the impact of sexual intercourse on the vaginal and underwear microbiome, as well as on patterns of microbial dispersal (Fig. 1A, B). We further explored whether such microbiome analyses, together with the current standard (i.e., semen detection), could provide additional trace evidence that would help corroborate activity level testimonies in forensic sexual assault cases. In addition, we aimed to increase the number of resolved sexual assault cases by facilitating investigations of trace biological evidence. Specifically, our analyses aim to improve semen detection, thereby enabling downstream, donor identification and the establishment of suspect profiles.

## Results

### Sexual intercourse causes temporal perturbations in the vaginal and underwear microbiome

**Fig. 2:**
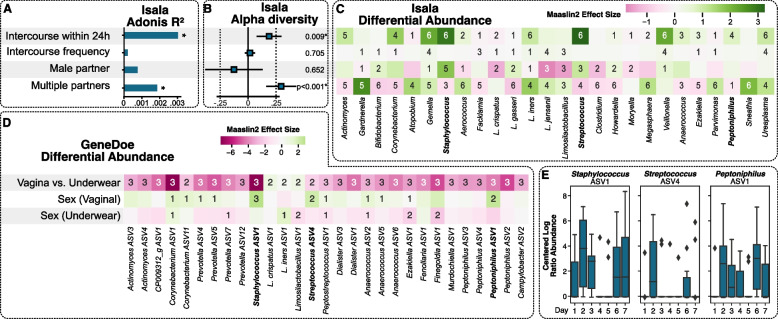
Effects of intercourse and partnerships on the vaginal microbiome from the Isala observational study (*n* = 3,043 participants, 439 of whom had recent sexual intercourse). Male partner (*n* = 2,317), female partner (*n* = 58), one partner (*n* = 2,319), multiple partners (*n* = 241), intercourse frequency is the average number of times per month sexual intercourse occurs. Panels left to right: **A **Effects on beta diversity (i.e., diversity between samples) * represents *p* < 0.05, **B **effects on alpha diversity (i.e., diversity within samples) * represents *p* < 0.05, and **C **effects on the relative abundances of specific bacteria. The number in each cell refers to the number of differential abundance methods that showed a significant effect for a given taxon. Taxa effect sizes are from Maaslin2. **D **GeneDoe differential abundance of ASVs between vaginal samples and underwear samples, and the effect of intercourse separately in vaginal and underwear samples (comparing days 1, 3, 4, 5, and 7 against days 2 and 6). The number in the cell represents the number of statistical methods (of Maaslin2, Limma, and a linear regression on the CLR-transformed abundances) for which the nominal *p* value was significant (*p* < 0.05). The effect sizes shown are from Maaslin2. **E **Centered-log ratio transformation (CLR) of potential driver taxa in vaginal samples over 7 days. *Peptoniphilus* ASV1, *Staphylococcus* ASV1 and *Streptococcus* ASV4 (right, left and middle panels, respectively)

We first investigated the impact of sexual intercourse on different features of the vaginal microbiome in our Isala cohort [20] (*n* = 3,043) (Fig. 1A). Isala is a large-scale citizen-science project [34] in Belgium and globally [35] with the aim to map the vaginal microbiome and its influencing factors (https://isala.be/en/). Here, we found that penile penetration in the last 24 h had a significant effect (R2 = 0.003, *p* = 0.008) on the beta and alpha (*p* = 0.009) diversity of the vaginal microbiome, resulting in a significantly greater relative abundance of several taxa, most notably *Streptococcus* and *Staphylococcus* (Fig. 2A–C). To further investigate the intrapersonal impact of penile penetration on the vaginal and underwear microbiome in a controlled longitudinal setting, we set up the GeneDoe project (Fig. 1B). Ten women collected seven identical pieces of cotton underwear, sampled their vaginas over the course of the 7-day intervention, and filled out surveys. They were asked to have sexual intercourse exclusively on the evenings of days 1 and 5. As a proxy for delayed sexual assault reporting, vaginal and underwear samples from days 6 and 7 were stored for 72 h at room temperature prior to analysis. Alpha diversity in specific but not all vaginal microbiomes (i.e., Shannon or within-sample diversity) increased after sexual intercourse. When we explored the differences in microbial composition (i.e., beta diversity) between successive days by calculating Bray‒Curtis distances, a greater effect was observed between pre- vs post-coital vaginal samples than between two pre-coital samples (Fig. 2A,B). We next went on to compare to a less-invasive sample, namely, underwear. Here, we found that while the vaginal microbiome observations were in line with the interpersonal observations made in the Isala study, they were less pronounced in the GeneDoe project’s underwear-derived microbiome profiles. This finding indicated that the greater diversity of underwear samples could overshadow pre- and post-coital differences (Figure S1). Nevertheless, underwear could capture the vaginal microbiome profile, as illustrated by the spatial community structure of vaginal swabs and underwear in a two-dimensional *t*-distributed stochastic neighbor embedding (t-SNE) space, Principal Coordinates Analysis (PCoA), and bar charts (Figure S2). The Bray‒Curtis distances (*p* < 0.01, permutation test), top taxa (most abundant), and abundance rankings of the top taxa (*p* < 0.01, permutation test) of the vaginal and underwear microbiome samples were similar.

We next investigated whether specific taxa exhibited a clearer pattern after sexual intercourse (Fig. 2D). Similarly, in the Isala study, we observed significantly greater relative abundances of ASVs pertaining to *Staphylococcus*,* Streptococcus*, and *Peptoniphilus* in post-coital vaginal samples (day 2). Like what was observed for diversity, these taxa were not altered in relative abundance in the underwear samples after sexual intercourse. We further looked at the centered-log ratio (CLR) transformation of the normalized abundance of these potential driver taxa calculated for each of the 7 days. This analysis revealed the significant presence of *Staphylococcus* ASV1, *Streptococcus* ASV4, and *Peptoniphilus* ASV1 in vaginal samples collected on the mornings after sexual intercourse (days 2 and 6) (Fig. 2E; results from alpha- and beta- association tests and differential abundance tests summarized in Table S1). Of note, 7 out of 10 participants received oral sex, but it was not registered whether this was before or after penile penetration. For *Streptococcus* ASV4, the presence of this ASV was short-term (only 1 day after sexual intercourse, i.e., days 2 and 6), while for *Peptoniphilus* ASV1, a greater abundance was observed over a longer time (i.e., up to 3 days after sexual intercourse, with days 3, 4, and 7 showing that for these bacteria), the vaginal ecosystem returned less quickly to a pre-coital state. We did find that the CLR of *Staphylococcus* ASV1 also increased the day after sexual intercourse and then decreased in subsequent days, followed by renewed introduction after the next sexual intercourse. Next, we assessed whether some of these taxa found in the vagina could originate from semen; to do so, we included data from a reference dataset of semen taxa from six unrelated individuals (Table S2). Two taxa, *Peptoniphilus* ASV1 and *Streptococcus* ASV4, were found in the semen samples and were also differentially abundant in the post-coital vaginal or underwear samples. These are potential indicator taxa for recent sexual intercourse with ejaculation.

### Vaginal microbiome can predict recent sexual intercourse

We subsequently conducted further analyses for two main purposes: (i) to gain a better understanding of the ecological drivers involved in vaginal microbiome changes after sexual intercourse and (ii) to predict recent sexual intercourse in a pool of vaginal samples without a priori information. To this end, we first used the Isala cross-sectional cohort dataset (Fig. 2A). Here, we trained a logistic elastic net regression classifier within a tenfold cross-validation loop and evaluated the performance of the classifier using the area under the curve (AUC = 0.90 ± 0.02; Table 1; Figure S3). AUC scores were significant, indicating that the model performance was significantly better than random (permutation test, *p* < 0.01; cfr. Methods). By selecting a posterior probability threshold that maximized the F1 scores within the cross-validation loops, we trained the classifier on the fully balanced dataset. Across all the samplings and folds, we observed a model accuracy of 0.71 ± 0.05, with a 0.81 ± 0.06 True Positive Rate and a 0.61 ± 0.08 True Negative Rate (Table 1). We further investigated the biological weight that particular taxa carried in this predictive model. Here, we found that taxa that contributed to a greater “after sexual intercourse or post-coital” posterior probability (i.e., having recent sexual intercourse; positive weights), among others, again included *Staphylococcus* (weight = 0.17), *Streptococcus* (weight = 0.08), and *Peptoniphilus* (weight = 0.03). We also identified several taxa (including *Limosilactobacillus*; weight = 0.006) that contributed to a lower “after sexual intercourse” posterior probability (i.e., not having recent sexual intercourse; negative weights) (Table S3).

**Table 1:**
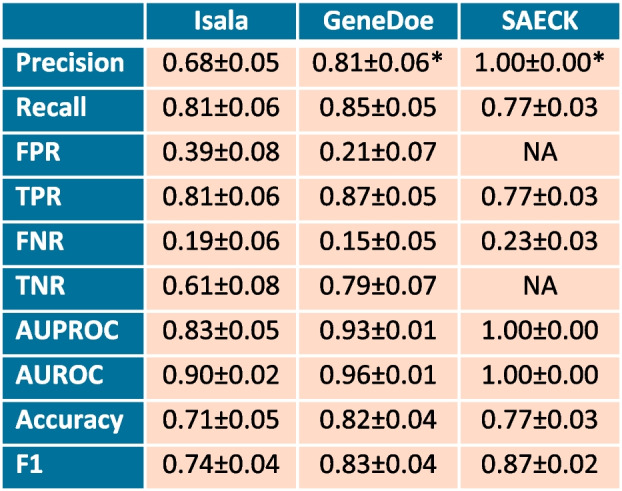
Balanced predictor evaluation metrics on the different datasets

*FPR* False Positive Rate, *TPR* True Positive Rate (self-reported sexual intercourse happened in the past 24 h), *FNR* False Negative Rate, *TNR* True Negative Rate (self-reported no sexual intercourse happened in the past 24 h), *AUPROC* Area Under the Precision-Recall Receiver Operating Characteristic, *AUROC* Area Under the Receiver Operating Characteristic, *NA* Not Applicable, *SAECK* Sexual Assault Evidence Collection Kit, there are no negative cases without sexual intercourse.

*Under the assumption that they had sexual intercourse with the actual result of semen found via PSA testing

**Fig. 3:**
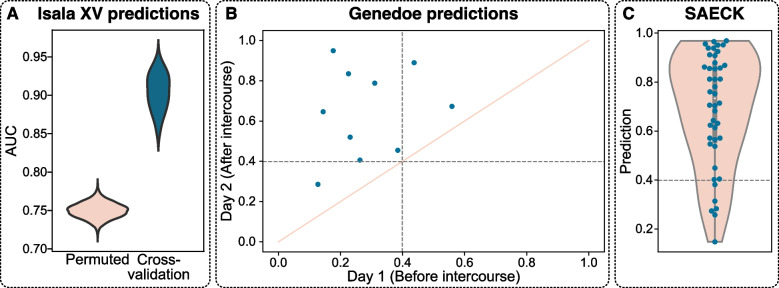
**A **Assessment of the predictive model in Isala: Significance testing of the Area Under the Receiver Operating Characteristic (ROC) Curve (AUC) indicated that the prediction performance was significantly better than random**.** The distributions of the AUCs calculated from the permuted labels are given in pink, and those calculated from the cross-validated machine learning model are given in blue. *Isala XV* refers to Isala cross validation **B** Prediction of sexual intercourse via the GeneDoe vaginal microbiome. The *x*-axis indicates the day 1 prediction (before sexual intercourse), and the *y*-axis indicates the day 2 prediction (after sexual intercourse). The diagonal line indicates the *y* = *x* line. Individuals above this line were assigned a higher score for their post-coital sample than for their pre-coital sample. **C **Prediction of sexual intercourse with 44 Sexual Assault Evidence Collection Kits (SAECKs; *n* = 88 vaginal samples). Panels **B** and **C** have a dotted line on the 0.399 prediction score threshold, determined through cross-validation (cfr. Methods)

When employing the predictive model on the GeneDoe dataset, we achieved an accuracy of 0.82 ± 0.04, a True Positive Rate of 0.87 ± 0.05 and a True Negative Rate of 0.79 ± 0.07. Moreover, for each individual participant, the “before sexual intercourse or pre-coital” vaginal samples had a significantly lower prediction score than the “after sexual intercourse or post-coital” samples (*p* = 0.002, Wilcoxon rank sum test; Fig. 3B; Figure S4). Finally, for potential application in the forensic field, we applied microbiome analysis to 44 samples obtained with Sexual Assault Evidence Collection Kits (SAECKs), for which we received legal permission to perform microbiome analysis (Figure S5). Applying our predictive model, we found that 36 of the 44 SAECKs analyzed were classified as post-coital (i.e., a prediction score of 0.399 or more, cfr. Methods), which according to our predictive model had an accuracy of 0.77 ± 0.03; these 36 samples were taken after sexual intercourse, and, likewise, the True Positive Rate was 0.77 ± 0.03; under the assumption that all the victims were telling the truth (Fig. 3C). We did not have information on whether penile penetration happened or not, how long ago or whether a condom was used. Under the assumption that PSA implies but not confirms intercourse, we performed prostate-specific antigen (PSA) testing for the detection of semen in the vaginal swabs. All 44 cases tested positive for PSA.

### Underwear as a complementary source of evidentiary value

**Fig. 4:**
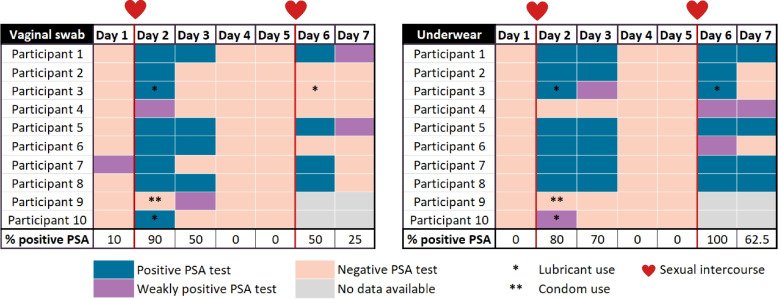
Semen detection in vaginal swabs and underwear with PSA tests. All participants (*n* = 10) wore seven pairs of underwear for seven consecutive days. Each pair was worn for 24 h and changed every morning when a vaginal swab was also taken. The red line and heart indicate that the night participants had sexual intercourse (evening of day 1 and day 5). The first five pairs of underwear and vaginal swabs were analyzed immediately after receipt, and the last two pairs of underwear and vaginal swabs were stored for 72 h at room temperature before analysis. Information about lubricant (*) or condom (**) use was included. For participants 9 and 10, samples from days 6 and 7 were not included in the analysis, as sexual intercourse did not occur on day 5. Positive PSA tests are indicated in blue, weakly positive PSA tests are indicated in purple, and negative PSA tests are indicated in light pink

Before including this novel microbiome tool in the forensic toolkit, we compared it to the gold standard used in sexual assault cases. Here, we simultaneously assessed current practices, as sampling from underwear is less invasive than vaginal swabs. To this end, we evaluated whether recent intercourse in our GeneDoe intervention dataset could be confirmed through the detection of semen based on PSA tests conducted on vaginal and underwear samples. On the morning after sexual intercourse, 8 out of the 10 underwear samples and 9 out of the 10 vaginal samples were positive for PSA (Fisher; *p* > 0.05) (Fig. 4). One of the negative results in each group could be attributed to condom use. After showering the next morning (all, except participant 6), participants changed underwear, and the next day, 7 out of 10 renewed underwear samples and 5 out of 10 vaginal samples still tested positive for PSA (*p* > 0.05). Over the next 2 consecutive days, all underwear and vaginal samples tested negative for PSA. On the evening of day 5, 8 out of the 10 participants again had penile penetrative sex with their partners. Both the underwear and vaginal samples were stored at room temperature for 72 h as proxies for delayed sexual assault analysis. All eight underwear samples tested positive for PSA, while this was the case for 4 out of the 8 vaginal samples (*p* > 0.05). At day 7 (approximately 34 h after sexual intercourse and showering), PSA tested positive in 5 of 8 (62.5%) underwear samples and in 2 of 8 (25%) vaginal samples. Taken together, these findingsè show that traces of semen can be detected in room temperature-stored underwear samples more frequently than in vaginal swabs, even after showering and changing underwear the day after sexual intercourse. This underscores the potential evidentiary value of underwear as a source for DNA analysis, which can support perpetrator(s) identification and physical evidence provision for trial in court. Nevertheless, each case requires individual assessment, as existing literature on the forensic relevance of underwear samples in current legal practice remains limited.

To address this gap, we also performed a retrospective descriptive study of 207 sexual assault cases and analyzed the relevant evidentiary value of the samples based on their forensic reports. We selected cases that included body fluid analyses of the evidence requested by the Prosecutor or Investigating Judge. Traces of semen were detected in 56.3% of the 151 analyzed SAECKs (equivalent to 23.9% of the 777 tested samples) through positive PSA results. Notably, the victim’s clothing (42.4% or *n* = 46/92) and objects such as condoms and bed sheets (70.6% or *n* = 12/17) produced more positive results, revealing a 1.8-fold greater likelihood of finding semen compared to samples derived from the body, such as vaginal swabs (24.1% or *n* = 32/133), similar to the findings of our GeneDoe study (Fig. 4). To further assess the ability to identify the donor of the semen and establish a suspect profile (sub-source level evidence), additional human DNA analysis was conducted for 66 of the 151 cases upon request from the Prosecutor or Investigating Judge. This analysis involved identifying a Y allele in the amelogenin marker to obtain a male DNA profile. Out of the 151 cases, an informative profile of the suspect could be generated and was sent to the National DNA database in 41 instances. Specifically, in 60% of these 41 cases, the male autosomal DNA profile was obtained from clothing and/or objects, indicating a greater success rate than from samples derived directly from the body. To further strengthen the evidence in forensic cases and increase their solvability, we suggest combining different analyses, for example, complementing PSA results for semen detection in underwear (source level evidence) with microbiome predictions of recent sexual intercourse (activity level evidence).

## Discussion

Our integrated study of multiple datasets, including an intervention study and recent sexual assault cases, explored the potential of a novel vaginal microbiome-based tool to predict recent sexual intercourse. Compared to the gold standard, we suggest complementing this current standard by including underwear evidence for better semen detection. Here, we explored the microbial interchange between sexual activity, the vaginal microbiome and underwear and between the vaginal and penile microbiomes, with relevance for forensic casework, human biology and reproduction.

Based on the data collected in this study, we built a sexual intercourse prediction model on vaginal microbiome profiles, which showed 77% accuracy and a True Positive Rate on actual forensic sexual assault cases (with no information available on the type of sexual assault, storage conditions, time between sexual assault and sampling or outcome of the case). These values were only slightly lower than the predictions in the GeneDoe samples from the controlled intervention (with all this information available). This predictive tool outperforms other machine learning tools in forensic microbiome research related to body fluid prediction [36–38], human identification [39], and post-mortem interval estimation [40, 41]. However, additional data could further improve this predictive tool, such as by accounting for hormonal fluctuations during the menstrual cycle and including larger cohorts with diverse ethnicities. Moreover, the type (such as orally received and/or given oral sex) and duration of sexual intercourse [42, 43] as well as the hygienic actions taken before and/or after sexual intercourse [43] are relevant questions that need further investigation as these may alter the microbial composition in ways that could impact the presence of specific taxa also commonly present in saliva, such as *Streptococcus* [44]. In addition, an important distinction is necessary regarding the time between the alleged event and the SAECK sampling. While the model was trained to detect intercourse that occurred within the last 24 h, we did not have access to the exact timing of the report or examination when selecting the kits. The 24 h benchmark was used as a criterion for the model based on the assumption that intercourse would be detectable within this timeframe. Therefore, the model is predicting only if sexual intercourse occured within the last 24 h, and for samples with a negative prediction (even in a model with 100% accuracy), it does not imply that sexual intercourse did not take place, merely that it was not in the last 24 h.We acknowledge that this timing uncertainty may impact classification accuracy in the SAECK samples and the model’s applicability in forensic settings.

In light of these considerations, the use and consequences of predictive models should also be carefully considered in their forensic, legal, and social contexts. In a court setting, it would serve to examine the veracity of the sexual assault accusation. Despite decades of feminist action to reduce the stigma associated with sexual assault, many victims still fear victim blaming, are hesitant to report their assault, and undergo full assessment [4]. A predictive model, even if it is to have near-perfect accuracy, can be used only as an indicator of further human DNA analysis and never as single evidence, as it could jeopardize trust in legal processes and lead to lower reporting rates. More specifically, in 77% of the real sexual assault cases investigated, our predictive model supported the testimony of a true victim (True Positive Rate), and a false sexual assault accusation could yield false testimony in 23% of the cases (False Negative Rate). Therefore, in the latter, a combination of evidence tools would benefit the case. While the rare phenomenon of false rape accusations [4] could be used to motivate such a predictive model, Hail-Jares *et al*. showed that false rape accusations are far less likely to result in the conviction of an innocent person (i.e., miscarriage of justice) [45]. To ensure the robustness of our approach and model, we recommend it to be validated by independent research groups on different populations to further examine its reliability. We therefore advocate for a predictive, probabilistic model of trace evidence on activity level, such as the one presented in this study, to be applied in sexual assault investigations as a complementary tool next to other traditional evidence, such as testimonies and human DNA analyses, while accounting for the error rates of individual tools when coming to a verdict. Moreover, microbiome research, innovation, and applications still warrant ethical holistic perspectives on guiding principles, regulations, and legislation [46].

While we did not have sufficient samples to construct a predictive model on the basis of underwear samples, our study did show that underwear might be useful for establishing a human DNA profile of the male suspect, considering its evidentiary value when assuming ejaculation and no use of condoms. Importantly, even after showering and changing underwear the next day, underwear still provided higher-frequency positive PSA test results (or semen detection) than vaginal swabs. Possible explanations could be that underwear is a more longitudinal sample, collecting vaginal discharge for 24 h, or that the neutrophilic leucocytes in the vaginal wall degrade spermatozoa through phagocytosis [47, 48], resulting in lower detection rates of PSA in vaginal samples. Similarly, drainage of semen from vaginal fluid could also lead to decreasing amounts of semen (and PSA) present in the vagina [48] as well as its dilution by vaginal fluid [49]. However, PSA has been shown to persist at detectable levels in the vagina up to 27 h postcoital [50] and spermatozoa even up to 5 [51] or 17 [48] days. However, due to the scope, design, and ethical constraints of the current study, we were unable to incorporate the semen of the participants’ partners; future research could explore the persistence and detectability of semen in a more controlled setting, including known timeframes of intercourse and the use of partners’ semen samples, to better understand the factors influencing PSA and spermatozoa degradation in both vaginal and underwear samples.

Nevertheless, our findings are not only relevant in fertility studies but also highly applicable to sexual assault investigations since 34.8% of victims reported bathing or showering after the assault prior to their examination [52]. Therefore, we propose extending the window for retrieving biological evidence, including cases where victims lost their original underwear. In addition to new underwear even after showering, our study showed that storing underwear for 72 h at room temperature generated results consistent with the outcomes of several in vitro studies [53–55]. Moreover, retrieving underwear from a victim is considered less invasive.

Although PSA tests are widely used in forensic settings, it is important to know that false positives can occur [53, 54, 56, 57]. One explanation is that PSA is also expressed in female periurethral glands when women are using contraceptives [55]. Nevertheless, our findings in a retrospective study were in line with our aforementioned PSA results, namely, that clothing and objects yielded more positive semen detection results than samples derived from the victim’s body. This could be explained by the fact that samples derived from the body often contain a significant amount of cell material from the victim [58] that can potentially lead to noise when generating DNA profiles from the assailant. It is important to note that DNA legislation, such as used in Belgium [59] and beyond, often prioritizes samples derived from the body, which is understandable considering its evidentiary value. In this specific study, we did not perform forensic DNA profiling on the underwear. Therefore, finding PSA on clothing does not necessarily imply that a probative DNA profile will be recoverable from these items. Additionally, the possibility of DNA (and PSA) transfer via contact must always be considered when evaluating samples from outside the body. Therefore, while our underwear results and retrospective conclusions suggest that clothing, particularly underwear, may provide valuable evidence in some cases, we acknowledge that further investigation is needed to assess its viability for generating probative DNA profiles. Based on our findings, we recommend that legislators carefully consider the circumstances of each specific case and the role of clothing and objects in their forensic investigations but temper expectations about the evidentiary value of these samples, particularly in cases where DNA profiling has not been performed.

Follow-up work is necessary to determine whether forensic microbiome profiling is suitable for routine case work, focusing specifically on its reproducibility, sensitivity, specificity, accuracy, detection limit, and error rates in relevant forensic settings, which need to be thoroughly assessed before it can be routinely implemented in forensic casework [18, 60]. Nevertheless, our study has made the first major steps toward a better understanding of relevant covariates related to sexual intercourse in a controlled environment with a microbial forensic perspective and applicability in the field.

## Conclusion

We showed that microbial dispersal occurs after recent sexual intercourse and that these signatures are captured by vaginal microbiome samples. Our findings demonstrate that forensic microbiome analyses could provide valuable information on recent sexual intercourse and could complement current techniques such as semen detection (at source level). We could accurately predict recent sexual intercourse based on specific driver taxa (*Streptococcus*,* Peptinophilus*, and *Staphylococcu*s) in vaginal microbiome swabs, despite heterogeneity in the responses (at activity level). This individualized response needs to be further investigated before this prediction tool can be applied to reliably detect sexual intercourse according to the scrutiny of forensic standards. Nevertheless, we show that semen detection in underwear has complementary value for documenting sexual intercourse, even after showering, changing into a new pair of underwear the next day and storing underwear at room temperature for 3 days. Taken together, the findings of this study highlight the evidentiary value of intimate clothing and microbial taxa analysis of vaginal swabs for sexual assault investigations, considering the circumstances for each case.

## Methods

### Isala cohort cross-sectional study

The Isala citizen-science project (https://isala.be/en/) was approved by the Ethical Committee of the Antwerp University Hospital/University of Antwerp (B300201942076) on November 18th, 2019, and registered online at clinicaltrials.gov with the unique identifier NCT04319536. A detailed study design and cohort information can be found in Lebeer *et al*. [8].

### GeneDoe study design and communication

Ten self-reported healthy, premenopausal women were recruited. Signed informed consent was obtained from all participants. This study was approved by the Ethical Committee of the Antwerp University Hospital/University of Antwerp (B3002021000066) on March 23rd, 2021. We aimed for an overlap in their menstrual cycles, allowing sampling on 8 consecutive days without any of the participants menstruating. To ensure that the study was carried out as optimally as possible, clear communication with our participants was essential for which mailings were used as the primary tool. Participants were first given some basic background information framing the study together with a first short summary of the goal of the study and what was expected from them. When participation was confirmed, communication proceeded via e-mail to obtain information about sizes for underwear, among other things. To ensure optimal procedures during the sampling days, a custom, creative brochure was designed. These brochures contained all the information with regard to optimal sampling. Participants were introduced to the term “swab” and guided through the self-swabbing procedure in a step-by-step, detailed protocol. Aside from the swabbing instructions, each participant provided a clear overview of which samples needed to be taken each day and of when and how delivery at our laboratory would occur.

### Sample collection with self-sampling kits

Ten participants were provided with a self-sampling kit containing seven pieces of underwear (Marks & Spencer London, 100% cotton, identical for every participant and washed uniformly as described below) and seven FLOQSwabs® in a sterile, dry 15-ml tube. Each day, participants were asked to (1) wear the (provided) underwear for 24 h; (2) take off the underwear of the day before; (3) put it in the provided paper bags; (4) fill in the paper bag label concerning the time of sexual intercourse, use of a condom, lubricant use, and partner’s ejaculation; (5) take a vaginal swab each morning; (6) take a shower if preferred; and (7) change into newly provided underwear for the next 24 h. On particular days, they were asked to have no sexual intercourse, while for two specific days (evenings of days 1 and 5), they were asked to have sexual intercourse with their partner before sleeping. On evenings of sexual intercourse, the participants were asked to put their underwear back on immediately afterwards and not to go to the toilet for 1 h; if they preferred, they could use a dry wipe. After each underwear change, the participants delivered their worn underwear packed in a paper bag, together with the swab of that morning, to the lab for immediate processing.

### Textile substrate

The underwear was washed before use. A standard washing program at 30 °C together with standard washing detergent and softener was used, after which the samples were air-dried for 24 h on disinfected drying racks in a room separated from any passage. Once dry, every pair of underwear was put separately in a bag. This whole process was performed using protective gear such as gloves and a mouth mask to avoid contamination.

### DNA extractions for the intervention study

Underwear was cut according to the seams of the vaginal-oriented part of the underwear (approximately 165 cm^2^). This piece was placed in a 50-ml tube with 15 ml of PBS (1x) using sterile tweezers. The tubes were shaken for 10 min (MO BIO Vortex Genie® 2 Vortex) before 10 min of centrifugation at 4 °C and 9000 rpm. Sterile tweezers were again used to wash every piece for maximum retrieval. Five hundred microliters of the remaining solution was then used for DNA extraction. The FLOQSwabs® were prepared for DNA extraction with the addition of 1.5 ml of CD1 solution to the swab. The swabs were incubated for 15 min at room temperature before being vortexed thoroughly for 15 s each. A total of 1.3 ml was then used for DNA extraction.

DNA was extracted using the DNeasy PowerSoil Pro Kit (Qiagen). The extraction itself was performed following the manufacturer’s manual, with adjustments to a starting volume of 500 µl and a 50 µl elution volume. For elution, 50 µL of C6 solution was added to the MB spin column and incubated for 5 min at room temperature. After the incubation, the spin column was centrifuged for 1 min at 15,000 × *g*. The flowthrough was subsequently added to the spin column and incubated for 3 min at room temperature before being centrifuged for 1 min at 15,000 × *g*. Afterwards, the extracted DNA was quantified via spectrophotometry (Take 3, BioTek) and fluorometry (Qubit 3.0 fluorometer, Life Technologies) and later stored at − 20°C.

### Sample selection of sexual assault kits

On 2 September 2019, ethical approval was obtained from the central ethical committee UAntwerp/UZA under EDGE455 to analyze Sexual Assault Evidence Collection Kits (SAECKs) from the forensic DNA lab of Antwerp University Hospital. Between 2003 and 2021, 1350 SAECKs were collected from sexual assault victims in the juridical district of Antwerp. By briefly reading the reports, a selection of SAECKs with the highest relevance was made for this study. We included only studies containing DNA extracts (by differential lysis with a QIAamp® DNA Mini Kit) for which both vaginal swabs and underwear extracts were available (*n* = 88). Finally, we selected SAECKs with the aim of obtaining an even distribution of DNA extracts over the years (i.e., a maximum of 5 SAECKs per year) (*n* = 44). Together, these findings led to the inclusion of total DNA extracts (the S and V fractions) in the form of vaginal swabs (Nswabs = 44, Nextracts = 88). All DNA extracts were stored at the Forensics DNA Laboratory of University Hospital Antwerp in 1.5-mL tubes at − 20°C. After searching for the selected SAECKs with their DNA extracts, the samples were thawed and vortexed. Finally, 20 μL was transferred to a new 1.5-mL tube for 16S rRNA sequencing. Given that these samples underwent standard forensic analyses with a focus on human DNA, they included both a potential victim DNA fraction (non-sperm fraction) and a potential suspect DNA fraction (sperm fraction) sample for each vaginal sample. Since the microbial compositions of both DNA fractions were similar, we pooled both fractions (Supplementary Figure S5).

### 16S rRNA amplicon sequencing

Amplification via PCR was performed for all samples, including negative controls (i.e., molecular-grade water and DNA extraction controls), using 96-well plates as described in Ahannach *et al*. [58]. According to the biomass of the samples, based on DNA quantification, different compositions of mastermix (MM) were prepared. Two-microliter samples with high biomasses were added to 14 µl of MM and 4 µl of 2.5 mM barcoded forward and reverse primer mix (2 µl forward and 2 µl reverse) in a single well. For the low-biomass samples, 5 µl was added to 11 µl of MM, and 4 µl of 2.5 µM barcoded forward and reverse primer mix was added (2 µl forward and 2 µl reverse). The primers used in this PCR were the 515F-806R primers, which were altered for dual-index paired-end sequencing, as described in Kozich et al. [61]. The sealed plates were centrifuged shortly before the PCR program was started. A 1% agarose gel was used to verify the resulting PCR products. The gel was loaded in each well with 1 µl of PCR product and 5 µl of loading dye (1:10) using a GeneRuler 1Kb Plus ladder (Thermo Scientific™). The gel was run for 30 min at 100 V to determine if the amplification was successful. Next, the PCR products were purified using Ampure XP (Agencourt) with a magnetic block following the manufacturer’s protocol. After purification, the DNA concentration was again measured using a Qubit 3.0 fluorometer, and the library was prepared by pooling equimolar concentrations of all the samples. Thirty microliters of the library was loaded with 6 µl of loading dye on a 0.8% agarose gel, together with a GeneRuler 1Kb Plus ladder. After running for 50 min at 60 V, gel extraction was performed using the NucleoSpin Gel and PCR Clean-up Kit (Macherey–Nagel) according to the manufacturer’s protocol. The final elution step of this kit was adjusted, whereas the DNA was eluted using 15 µl of the provided elution buffer and subsequently reloaded on the column for increased yield. The concentration was measured using a Qubit 3.0 fluorometer to dilute the library to 2 nM, which was again confirmed with a final Qubit 3.0 fluorometer. Next, 0.2 N NaOH (Illumina, San Diego, California, USA) was used for denaturing the library, which was subsequently diluted to 6 pM and spiked with 10% Phix control DNA (Illumina). The V4 region of the 16S rRNA gene was sequenced using dual-index paired-end sequencing (2 × 250) with a MiSeq Desktop sequencer (Illumina).

### Forensic presumptive and confirmatory testing of semen via underwear and vaginal swabs

First, pictures were taken from each piece of underwear, and vaginal secretion amounts were subjectively described (i.e., little or average). Next, an alternative light source (Crime-Lite® 2, 420–470 nm BLEU, Foster & Freeman) was used to detect the fluorescence of possible semen stains. Detection with Crime-lite served as an indication to cut a small piece (approximately 0.5 cm^2^) of the underwear and the tip of the vaginal swab for PSA testing (PSA-CHECK-1, Veda-Lab). Then, 300 µl of 0.9% NaCl was added to the the piece of underwear, which were vortexed thoroughly and incubated for 10 min at room temperature. After incubation, 225 µl was added to the sample well, and the results were interpreted after 10 min. In case of suspicion of the presence of blood due to reddish spots after visual inspection with natural light and Crime-light®, an additional blood test (BLUESTAR® OBTI) was performed on the underwear. A small piece (approximately 0.5 cm^2^) of underwear was added to 20 µl of Tris buffer (pH 7.8), vortexed thoroughly and incubated for 5 min at room temperature. Then, 120 µl was added to the sample well, and the results were obtained after 5 min of incubation.

### Semen sample collection and processing dataset

The FORmics project at the Institute of Forensic Medicine in Switzerland was approved by CEBES, the Ethics Review Board at the University of Zurich, and received the following identifier 2021-11c. Semen samples were self-collected by 6 male participants. For each of these, two biological replicates were generated by depositing 50 μL of sample on a swab, resulting in a total of 12 samples. DNA was extracted with the QIAamp BiOstic Bacteremia DNA Kit (Qiagen), and library preparation for the V3–V5 16S rRNA gene region was carried out with the F357 and R926 primers (detailed in Table S4). The volume of the library preparation PCR mixture was 25 μL, and the mixture contained 2X Phusion Hot Start II High-Fidelity PCR Master Mix. The reaction mixture contained 1 μL of DNA, 12.5 μL of 2X Phusion Hot Start II High-Fidelity PCR Master Mix, 1.25 μL of forward primer, 1.25 μL of reverse primer, and 9 μL of PCR-grade water. A PCR negative control was included where the reaction well contained 1 μL of PCR-grade water. The thermocycling conditions for the amplicon PCR were as follows: 30 s at 98 °C for the initial incubation followed by denaturation; 32 cycles of 10 s at 98 °C and annealing for 30 s at 50 °C for the V3–V5 region; extension for 25 s at 72 °C; a final extension for 10 min at 72 °C; and hold at 4°C. The integrity of the amplicon PCR products was checked using 1.5% agarose gel electrophoresis, where the gel was loaded with 3 μL of PCR product, 2.5 μL of distilled water, and 3 μL of dye. The PCR products were purified using magnetic beads according to the manufacturer’s protocol (AMPure XP, Beckman Coulter) at a ratio of 0.8-fold the volume of AMPure XP beads per volume of PCR product. Next, the purified amplicon DNA was dual-indexed using the Nextera XT Index Kit. PCR was performed in a total volume of 25 μL with 5 μL of purified PCR product, 12.5 μL of 2X Phusion Hot Start II High-Fidelity PCR Master Mix, 2.5 μL of Index 1, 2.5 μL of Index 2, and 2.5 μL of molecular-grade water. The thermocycling conditions for the index PCR were 30 s at 98 °C for initial incubation, followed by 12 cycles of denaturation for 10 s at 98 °C, annealing for 30 s at 55 °C, extension for 20 s at 72 °C, a final extension for 5 min at 72 °C and holding at 4°C. The indexed PCR products were purified with AMPure XP beads at a ratio of 0.6-fold the volume of AMPure XP beads per volume of PCR product. The library products were quantified using a Spark 10 M Multimode Microplate Reader (Tecan) and a Qubit dsDNA BR assay (Thermo Scientific). Libraries were normalized to 3.03 nM, pooled, denatured with 0.2 M NaOH, and diluted to a final concentration of 20 pM. A PhiX sequencing control (Illumina) was used for sequencing. The PhiX library was prepared by diluting the 10 nM PhiX library (2 µl) with 10 nM Tris–Cl, pH 8.5 (3 µl), to 4 nM. Then, 5 µl of the 4 nM PhiX library was denatured with 5 µl of 0.2 N NaOH. The denatured PhiX was diluted to 20 pM by adding 990 µl of prechilled HT1 solution to 10 µl of the PhiX library. Sequencing was carried out on an Illumina MiSeq platform (Illumina, Inc., Hayward CA, USA) with the MiSeq Reagent Kit V3, generating paired-end reads of 2 × 300 bp. The raw reads were trimmed with Cutadapt v3.5 and quality filtered and denoised with the DADA2 pipeline [version 1.18.0] with the following parameters. (maxN = 0, maxEE = c(4,6), truncQ = 2), resulting in an ASV abundance table for 12 semen samples.

### Retrospective study

To study the prevalence of risk factors, medico-legal examinations and SAECK analysis, medicolegal findings were correlated with DNA analysis results, providing novel information that could be used to design guidelines and/or suggestions to optimize the current approach in cases of sexual assault. A retrospective descriptive case study (Local KULeuven ethics committee [mp13636]) of 207 sexual assault cases was performed by the Laboratory of Forensic Genetics [UZ Leuven] from 2012 until 2016. After excluding 56 cases, 151 cases were left for analysis. The exclusion criteria were as follows: a forensic analysis (biological trace evidence examination and DNA profiling) had to have been requested by the court; the case was a sexual assault; and PSA tests for the presence of semen had to be performed. The remaining 151 cases were studied in more depth with regard to background information, time and date, medical examination, and DNA analysis.

### Data analysis

The abovementioned results were analyzed with R-studio (version 1.4.1106) using the in-house-developed tidyamplicons package (github.com/Swittouck/tidyamplicons) and the tidyverse package for analyzing diversity between samples (beta diversity) and within samples (alpha diversity). For alpha diversity, Shannon diversity was used as a measure of entropy. The Bray‒Curtis distance was used to measure the beta diversity between samples. With respect to the Isala dataset, we tested the association between reported sexual intercourse in the last 24 h and no intercourse, adjusting for age, recent antibiotic usage, and technical covariates, using six different differential abundance methods: ALDeX2 [62], ANCOM-BC [63], DESeq2 [64], Limma (with voom transformation) [65], a linear model fit on the Centered Log Ratio transformation [66], and Maaslin2 [67]. Each factor was adjusted for technical confounders, including run and library size, and adjusted for age and antibiotic usage in the last 3 months. The Benjamini–Hochberg procedure was used to control the False Discovery Rate and correct *p* values for multiple testing. In the GeneDoe dataset, we added a random effect per participant to pair samples; as a result, only the Limma, CLR, and Maaslin2 methods could be applied, as the others do not support random effects. For GeneDoe, the reported *p* values are nominal and were not corrected for multiple testing because the sample size was too small and we lacked power. All these methods were implemented in a unified interface in the multidiffabundance R package (https://github.com/thiesgehrmann/multidiffabundance).

To classify samples into post-coital and non-post-coital samples, we needed to create a balanced dataset. With 410 individuals who had reported intercourse within the last 24 h, we created a balanced dataset by sampling an equal number of individuals who indicated that they had not had intercourse within the last 24 h. To ensure that performance was not based on a fortuitous sampling, we repeated this process 10 times. Within each downsampling, we performed a tenfold cross-validation. In the cross-validation loop, we trained a logistic elastic net classifier with a fixed alpha (penalty weight) of 0.5. An inner cross-validation loop was used for hyperparameter optimization (L1 and L2 mixing ratios). We evaluated the performance of the classifier with respect to the area under the curve (AUC) and determined whether these scores were significantly better than random with a permutation test in which the posterior probabilities were permuted and the area under the curve (AUC) recalculated. Within each fold, we selected the posterior probability (post-coital score) threshold that resulted in the highest F1 score within each fold. For the final model, we used the average threshold across the 10 samplings and the 10 folds of the cross-validation, resulting in a threshold of 0.399. Using the same downsampling sets as before, we trained 10 models on the fully balanced datasets. Finally, we tested these models against the GeneDoe and sexual assault kit datasets, and we report the performance as the average and standard deviation of the predictions across all repeats. The weights reported in supplementary Table S3 reflect the average weights of the full models across the 10 down-samplings. In addition, ASVs from the GeneDoe study (for which only the V4 region was sequenced, 2 × 250 bp) were effectively matched within the broader ASVs (Table S2) from the Swiss seminal microbiome (for which the V3–V5 hypervariable regions were sequenced, 2 × 300 bp) using exact substring matching.

## Supplementary Information

Supplementary Material 1: Figure S1. The temporal effects of sexual intercourse on the microbiome of vaginal swabs as analyzed with (A) Shannon diversity of underwear and vaginal swabs and (B) Bray‒Curtis distance in underwear and vaginal samples across time for each participant (each day compared to the day before). Profiles are min–max normalized within each participant, meaning that the scores are scaled such that the lowest value in the personal profile is 0 and the highest is 1. The scale is indicated in blue on the right. *Participants 9 and 10 did not have sexual intercourse on day 5. (C) Bray‒Curtis distance in vaginal (left) and underwear (right) samples across time for all participants (each day compared to the day before). Figure S2. Microbial structure of vaginal samples and underwear in the t-SNE space (row 1) and the PCOA space (row 2), colored by (A) type of sample, (B) Shannon entropy, (C) most abundant taxa, (D) secondmost abundant (sub)genus, and (E) largest relative abundance level of the most dominant taxon. Taxonomic classification of the microbial composition of (F) vaginal samples and (G) underwear. The most abundant (sub)genera are visualized. Figure S3. Cross-validation of true and false positive rates. The ROC curve per fold in the cross-validation (left panel) and the precision–recall curve (right panel) are shown. Figure S4. Prediction of coital status via the elastic net for the vaginal microbiome (days 1 to 7) in the GeneDoe dataset. Points called post-coital are in pink (darker = higher prediction), and points in blue are pre-coital (darker = lower prediction). Asterisks indicate points that passed the threshold (indicated in the color bar). Participants 9 and 10 did not have sexual intercourse on day 5. Figure S5. Prediction of the coital status of 44 sexual assault evidence collection kits (SAECKs or rapekits) (*n* = 88 vaginal samples). Given that these samples underwent standard forensic analyses with a focus on human DNA, they included a victim DNA fraction (yellow = VF) and a suspect DNA fraction (blue = SF) for each vaginal sample. Since their microbial compositions were similar, we pooled the samples (green = full). Samples from the same participant are connected with a line. Table S1. The results of the alpha- and beta-diversity association tests and differential abundance test from the Isala and GeneDoe dataset. Separate Excel tables. Table S2. Matched ASV sequences from the reference dataset of semen taxa from six different individuals. Separate Excel tables. Table S3. Weight statistics of the prediction model. Negative weights refer to not having recent sexual intercourse, while positive weights refer to having recent sexual intercourse. Table S4. The primer sequences, including 1–3 bp (Ns) and Illumina adapter overhang nucleotide sequences, are shown in the table below.

## Data Availability

Sequence data that support the findings of this study have been deposited in the European Nucleotide Archive with the primary accession codes PRJEB50407 and PRJEB75041.
