# Supplementary material for: Microbial and seminal traces of sexual intercourse and forensic implications
Source: Microbiome. 2025 Nov 20;13:237. doi: 10.1186/s40168-025-02268-7 (PMC12632010; doi:10.1186/s40168-025-02268-7)
Supplement: Supplementary file 2 — Supplementary Material 1: Figure S1. The temporal effects of sexual intercourse on the microbiome of vaginal swabs as analyzed with (A) Shannon diversity of underwear and vaginal swabs and (B) Bray‒Curtis distance in underwear and vaginal samples across time for each participant (each day compared to the day before). Profiles are min–max normalized within each participant, meaning that the scores are scaled such that the lowest value in the personal profile is 0 and the highest is 1. The scale is indicated in blue on the right. *Participants 9 and 10 did not have sexual intercourse on day 5. (C) Bray‒Curtis distance in vaginal (left) and underwear (right) samples across time for all participants (each day compared to the day before). Figure S2. Microbial structure of vaginal samples and underwear in the t-SNE space (row 1) and the PCOA space (row 2), colored by (A) type of sample, (B) Shannon entropy, (C) most abundant taxa, (D) secondmost abundant (sub)genus, and (E) largest relative abundance level of the most dominant taxon. Taxonomic classification of the microbial composition of (F) vaginal samples and (G) underwear. The most abundant (sub)genera are visualized. Figure S3. Cross-validation of true and false positive rates. The ROC curve per fold in the cross-validation (left panel) and the precision–recall curve (right panel) are shown. Figure S4. Prediction of coital status via the elastic net for the vaginal microbiome (days 1 to 7) in the GeneDoe dataset. Points called post-coital are in pink (darker = higher prediction), and points in blue are pre-coital (darker = lower prediction). Asterisks indicate points that passed the threshold (indicated in the color bar). Participants 9 and 10 did not have sexual intercourse on day 5. Figure S5. Prediction of the coital status of 44 sexual assault evidence collection kits (SAECKs or rapekits) (n = 88 vaginal samples). Given that these samples underwent standard forensic analyses with a focus on human DNA, they inclu [file 40168_2025_2268_MOESM1_ESM.zip › Supplementary/Ahannach_et_al_Microbiome_supplementary_files_20250214.docx]

**Supplementary files***Microbial and seminal traces of sexual intercourse and forensic implications*

**Supplementary Figures**

**
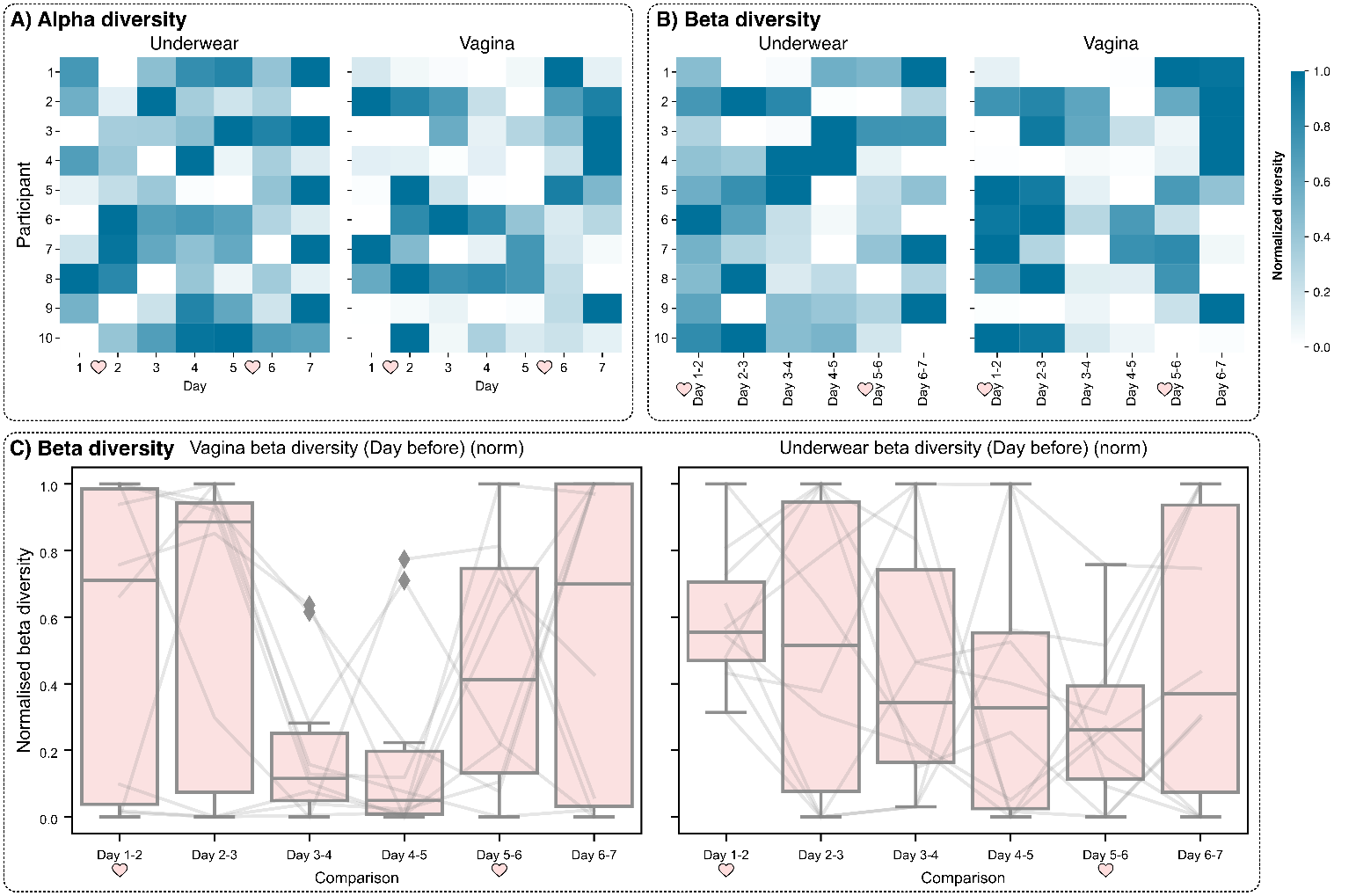
**

**Figure S1: The temporal effects of sexual intercourse on the microbiome of vaginal swabs as analyzed with** (A) Shannon diversity of underwear and vaginal swabs and (B) Bray‒Curtis distance in underwear and vaginal samples across time for each participant (each day compared to the day before). Profiles are min–max normalized within each participant, meaning that the scores are scaled such that the lowest value in the personal profile is 0 and the highest is 1. The scale is indicated in blue on the right. *Participants 9 and 10 did not have sexual intercourse on day 5. (C) Bray‒Curtis distance in vaginal (left) and underwear (right) samples across time for all participants (each day compared to the day before).

**
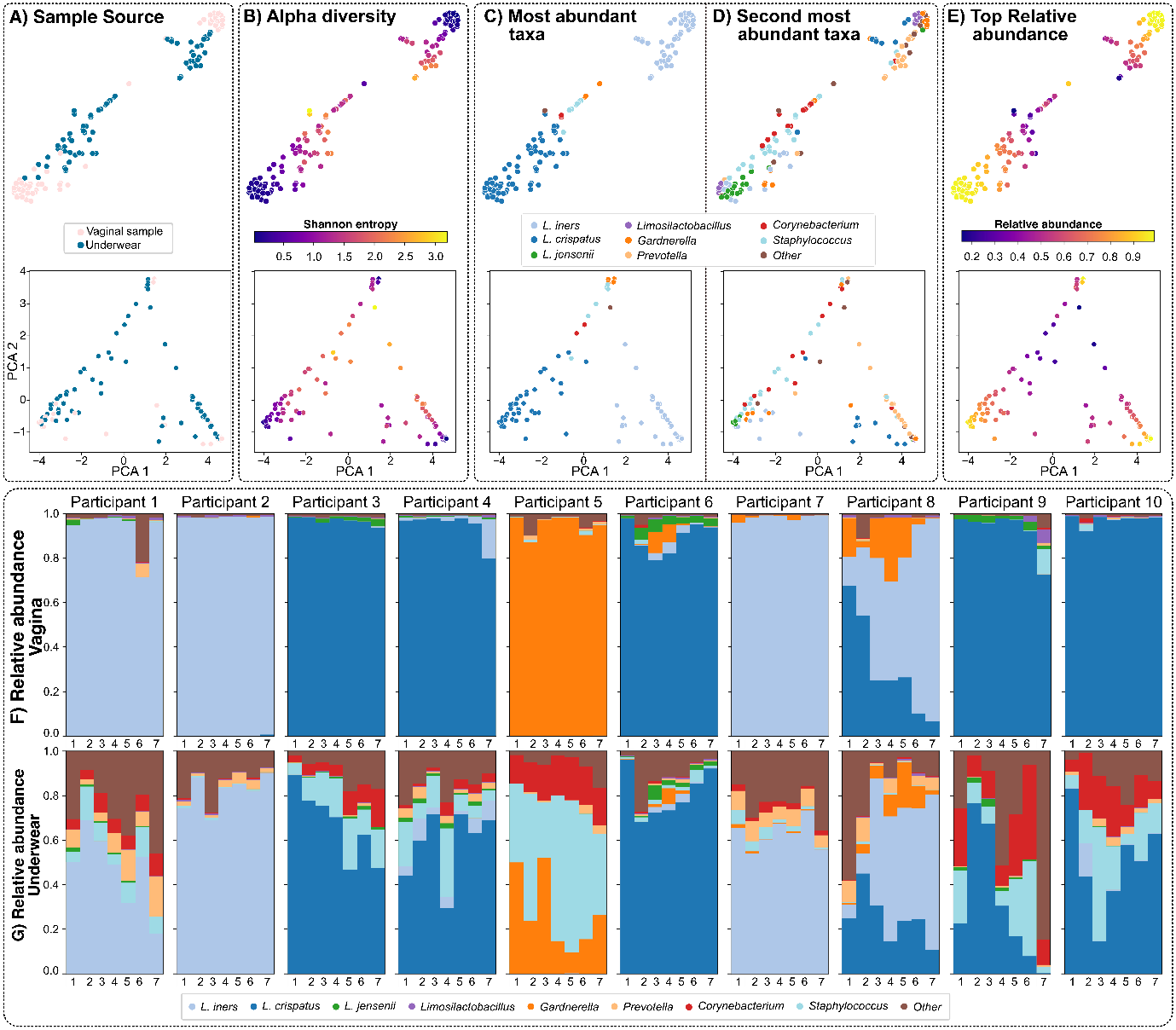
**

**Figure S2: Microbial structure of vaginal samples and underwear in the t-SNE space (row 1) and the PCOA space (row 2)**, colored by (A) type of sample, (B) Shannon entropy, (C) most abundant taxa, (D) secondmost abundant (sub)genus, and (E) largest relative abundance level of the most dominant taxon. **Taxonomic classification of the microbial composition of (F) vaginal samples and (G) underwear**. The most abundant (sub)genera are visualized.

**
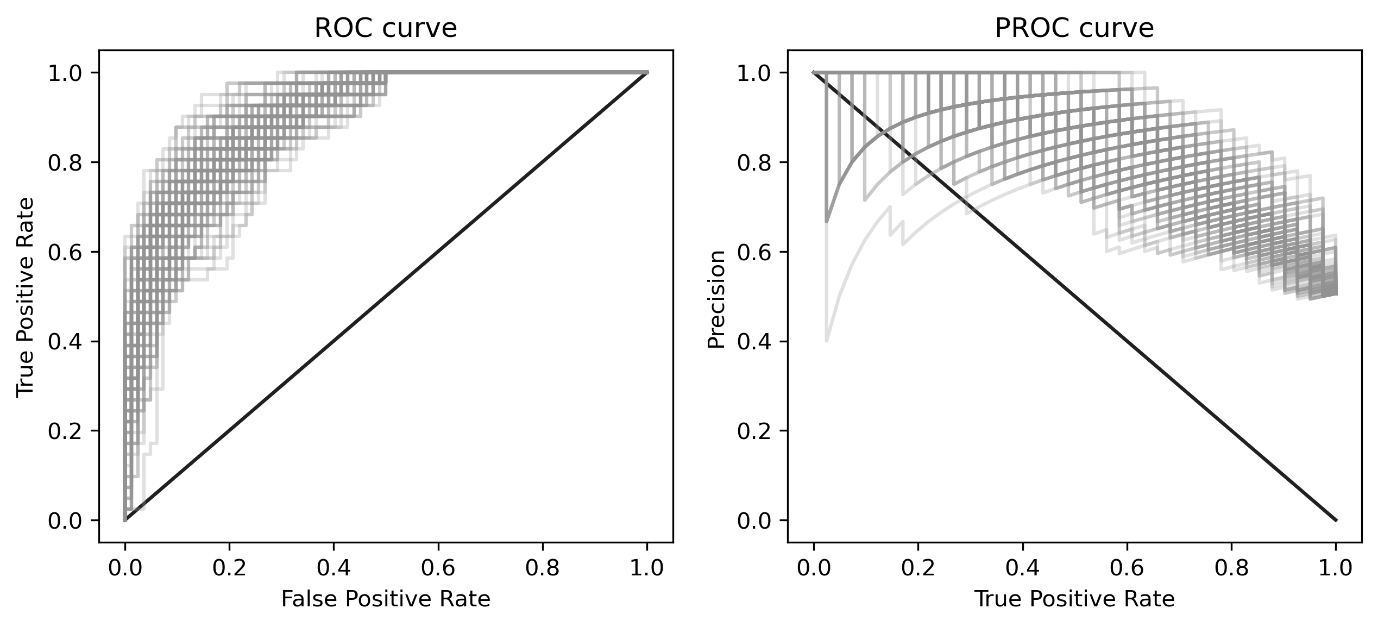
**

**Figure S3: Cross-validation of true and false positive rates.** The ROC curve per fold in the cross-validation (left panel) and the precision–recall curve (right panel) are shown.


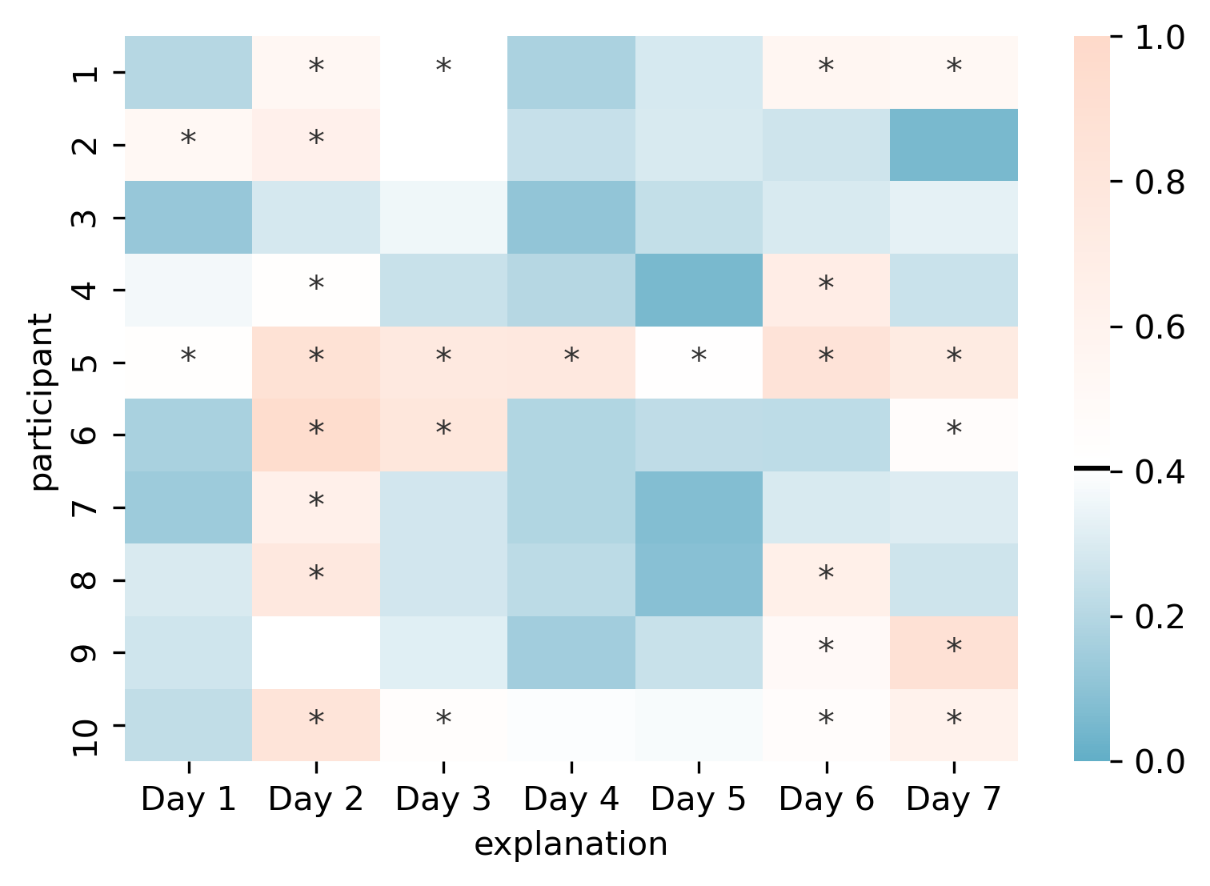


**Figure S4: Prediction of coital status via the elastic net** for the vaginal microbiome (days 1 to 7) in the GeneDoe dataset. Points called post-coital are in pink (darker = higher prediction), and points in blue are pre-coital (darker = lower prediction). Asterisks indicate points that passed the threshold (indicated in the color bar). Participants 9 and 10 did not have sexual intercourse on day 5.


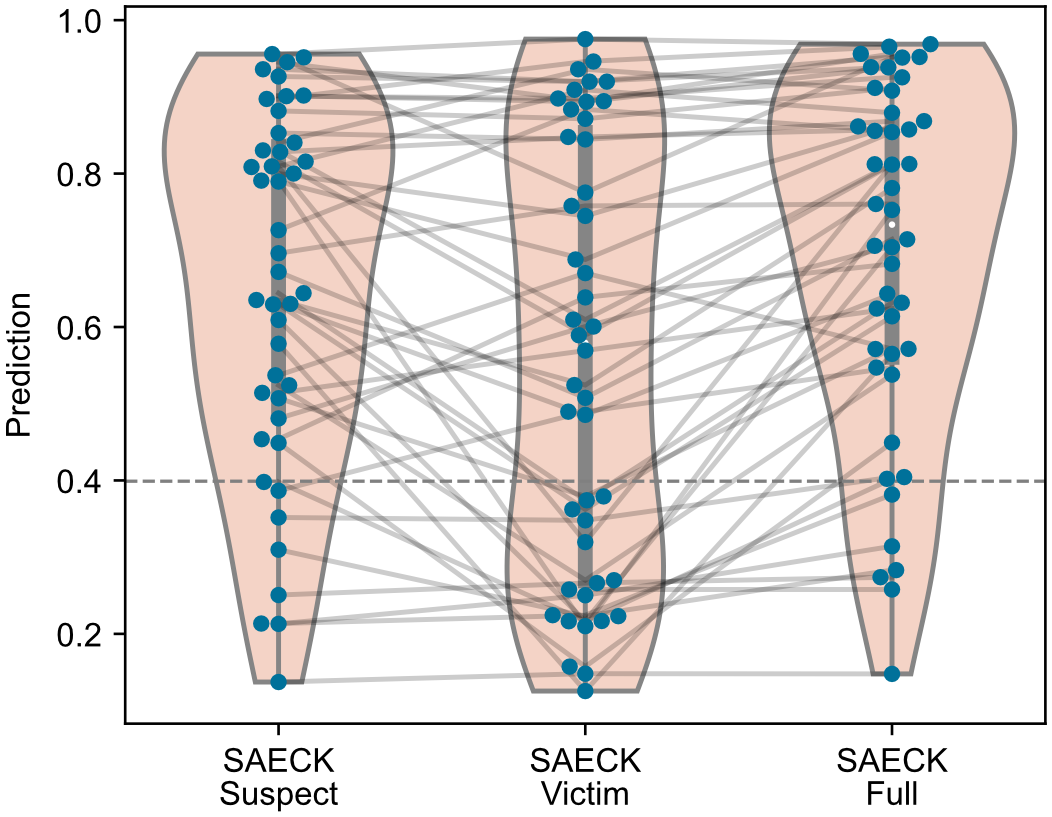


**Figure S5: Prediction of the coital status of 44 sexual assault evidence collection kits (SAECKs or rapekits) (n = 88 vaginal samples).** Given that these samples underwent standard forensic analyses with a focus on human DNA, they included a victim DNA fraction (yellow = VF) and a suspect DNA fraction (blue = SF) for each vaginal sample. Since their microbial compositions were similar, we pooled the samples (green = full). Samples from the same participant are connected with a line.

**Supplementary Tables**

**Table S1: The results of the alpha- and beta-diversity association tests and differential abundance test from the Isala and GeneDoe dataset**

***Separate Excel tables***

**Table S2: Matched ASV sequences from the reference dataset of semen taxa from six different individuals**

***Separate Excel tables***

**Table S3: Weight statistics of the prediction model.** Negative weights refer to not having recent sexual intercourse, while positive weights refer to having recent sexual intercourse.

**Table S4: The primer sequences,** including 1-3 bp (Ns) and Illumina adapter overhang nucleotide sequences, are shown in the table below.

| Region | Name | 5’-3’ |
| --- | --- | --- |
| V3-V5 | F357_n0 | TCGTCGGCAGCGTCAGATGTGTATAAGAGACAGT**CCTACGGGAGGCAGCAG** |
| V3-V5 | F357_n1 | TCGTCGGCAGCGTCAGATGTGTATAAGAGACAGNT**CCTACGGGAGGCAGCAG** |
| V3-V5 | F357_n2 | TCGTCGGCAGCGTCAGATGTGTATAAGAGACAGNNT**CCTACGGGAGGCAGCAG** |
| V3-V5 | R926_n0 | GTCTCGTGGGCTCGGAGATGTGTATAAGAGACAGT**CCGTCAATTCMTTTRAGT** |
| V3-V5 | R926_n1 | GTCTCGTGGGCTCGGAGATGTGTATAAGAGACAGNT**CCGTCAATTCMTTTRAGT** |
| V3-V5 | R926_n2 | GTCTCGTGGGCTCGGAGATGTGTATAAGAGACAGNNT**CCGTCAATTCMTTTRAGT** |
